# Amyloid β and tau pathology in brains of aged pinniped species (sea lion, seal, and walrus)

**DOI:** 10.1186/s40478-020-01104-3

**Published:** 2021-01-07

**Authors:** Yuta Takaichi, James K. Chambers, Kei Takahashi, Yoshiyuki Soeda, Riki Koike, Etsuko Katsumata, Chiaki Kita, Fuko Matsuda, Makoto Haritani, Akihiko Takashima, Hiroyuki Nakayama, Kazuyuki Uchida

**Affiliations:** 1 Laboratory of Veterinary Pathology, Graduate School of Agricultural and Life Sciences, The University of Tokyo, Tokyo, 113-8657 Japan; 2 Department of Life Science, Faculty of Science, Gakushuin University, Tokyo, Japan; 3 Kamogawa Seaworld, Chiba, Japan; 4 Shikoku Cytopathological Laboratory, Kagawa, Japan; 5 Laboratory of Theriogenology, Graduate School of Agricultural and Life Sciences, The University of Tokyo, Tokyo, Japan; 6 Environmental Science for Sustainable Development, The University of Tokyo, Tokyo, Japan

**Keywords:** Alzheimer’s disease, Amyloid β, GSK-3β, Tau, Pinniped, Neurodegeneration

## Abstract

Alzheimer’s disease (AD) is characterized by the accumulation of amyloid-β (Aβ) as senile plaques and cerebral amyloid angiopathy, and hyperphosphorylated tau (hp-tau) as neurofibrillary tangles in the brain. The AD-related pathology has been reported in several non-human animals, and most animals develop only the Aβ or tau pathology. We herein describe the Aβ and hp-tau pathology in the brains of aged pinniped species (seal, sea lion, and walrus). Molecular analyses revealed that the sequence of pinniped Aβ was identical to that of human Aβ. Histopathological examinations detected argyrophilic plaques composed of Aβ associated with dystrophic neurites in the cerebral cortex of aged pinnipeds. Astrogliosis and microglial infiltration were identified around Aβ plaques. Aβ deposits were observed in the blood vessel walls of the meninges and cerebrum. Pinniped tau protein was physiologically subjected to alternative splicing at exons 2, 3, and 10, and presented as five isoforms: two 3-repeat tau isoforms (1N3R, 2N3R) and three 4-repeat tau isoforms (0N4R, 1N4R, 2N4R); 0N3R tau isoform was absent. Histopathological examinations revealed argyrophilic fibrillar aggregates composed of hp-tau in the neuronal somata and neurites of aged pinniped brains. Few hp-tau aggregates were found in oligodendrocytes and microglia. Biochemically, hp-tau of the 3-repeat and 4-repeat isoforms was detected in brain sarkosyl-insoluble fractions. Aβ and hp-tau both predominantly accumulated in the neocortex, particularly the frontal cortex. Furthermore, the activation of GSK-3β was detected within cells containing hp-tau aggregates, and activated GSK-3β was strongly expressed in cases with severe hp-tau pathologies. The present results suggest that, in association with Aβ deposition, the activation of GSK-3β contributes to hp-tau accumulation in pinniped brains. Here, we report that pinniped species naturally accumulate Aβ and tau with aging, similar to the human AD pathology.

## Background

Alzheimer’s disease (AD) is the most prevalent age-related neurodegenerative disorder and is characterized by the pathological aggregation of the amyloid-β (Aβ) and hyperphosphorylated tau (hp-tau) proteins in the form of senile plaques (SPs) and neurofibrillary tangles (NFTs), respectively [1]. The accumulation of Aβ in the blood vessels of the brain, a condition known as cerebral amyloid angiopathy (CAA) [2], is also detected in more than 80% of patients with AD [3]. Humans appear to be uniquely susceptible to AD, potentially due to genetic differences, changes in cerebral structures and functions during evolution, and an increased lifespan [4–7]. In the ‘‘amyloid hypothesis’’, the most acknowledged explanation for the pathogenesis of AD, the age-dependent accumulation of fibrillar insoluble Aβ peptides in the brain is considered to be the central and triggering event in AD pathology [8]. Based on this hypothesis, various transgenic mouse models that produce human Aβ beyond physiological levels have been generated and exhibit the massive formation of SPs. However, they fail to develop NFTs and neuronal loss unless mutant tau is simultaneously introduced [9].

While AD appears to be a human-specific disease, age-dependent SP formation has been reported in several non-human primates, including chimpanzees [10], orangutans [11], and gorillas [12]. The concomitant pathology with the formation of a small amount of NFTs was found in chimpanzees [13, 14] and rhesus macaques [15], while the oligodendroglial tau pathology was also detected in cynomolgus monkeys [16]. Therefore, an AD-like pathology may occur during aging in primates. In contrast, non-primate animals, particularly Carnivora species, have exhibited species-specific patterns of Aβ and hp-tau accumulation (Table 1). In the suborder Caniformia, aged dogs [17, 18] and bears [19] developed SPs in their brains, but not NFTs, even in the oldest subjects, except for one wolverine [20]. On the other hand, Feliformia species, such as cats [21, 22], leopard cats [23], and cheetahs [24], exhibit NFTs without SP formation, although small granular deposits of Aβ were detected in the cerebral cortex.

**Table 1:** Occurrence of senile plaques (SP) and neurofibrillary tangles (NFT) in carnivora species

Suborder	Infraorder	Superfamily	Family	Species	SP	NFT
Caniformia	Cynoidea		Canidae	Domestic dog (*Canis lupus familiaris*)	Yes	No
	Arctoidea	Pinnipedia	Phocidae	Harbor seal (*Phoca largha*, *Phoca vitulina*)	Yes	Yes
			Otariidae	Northern sea lion (*Eumetopias jubatus*)	Yes	Yes
				California sea lion (*Zalophus californianus*)	Yes	Yes
				Australian sea lion (*Neophoca cinerea*)	Yes	Yes
			Odobenidae	Walrus (*Odobenus rosmarus*)	Yes	Yes
		Musteloidea	Mustelidae	Wolverine (*Gulo gulo*)	Yes	Yes
		Ursoidea	Ursidae	American black bear (*Ursus americanus*)	Yes	No
Feliformia		Feloidea	Felidae	Domestic cat (*Felis silvestris catus*)	No	Yes
				Leopard cat (*Prionailurus bengalensis*)	No	Yes
				Cheetah (*Acinonyx jubatus*)	No	Yes

Pinnipeds are semiaquatic carnivorans that spend most of their lives in water, and use coastal terrestrial environments and ice packs to breed, molt, and rest. They are currently classified into three families: Phocidae (seals), Otariidae (fur seals and sea lions), and Odobenidae (walruses). They may have evolved within a group of arctoid carnivores in the Pacific Ocean 27 million years ago. Although their origin is a matter of discussion, they are currently regarded as a monophyletic group [25]. One case study confirmed SP formation and CAA, but not NFTs, in the brain of a 30-year-old California sea lion (*Zalophus californianus*) [26]. In the present study, we performed comprehensive genetic, biochemical, and pathological analyses of the brains of young to old pinniped species (aged between 0 and 35 years). The results obtained showed that aged pinniped species exhibited the concomitant accumulation of Aβ and hp-tau in their brains. Pinniped brains produced Aβ with an amino acid sequence that was identical to that of humans, developed SPs in the cerebral cortex, and exhibited CAA. Their brains expressed five isoforms of tau (1N3R, 2N3R, 0N4R, 1N4R, and 2N4R), and formed NFTs in the neuronal somata and neurites of the cerebral cortex. We herein report our results on the Aβ and tau pathology in the brains of pinniped species and discuss the relationship between the accumulation of Aβ and hp-tau in consideration of comparative biology.

## Methods

### Animals

Ten pinniped brain tissues were examined (Table 2). All samples were obtained through routine necropsies performed at the Laboratory of Veterinary Pathology, the University of Tokyo. All procedures were conducted according to the institutional regulations for animal research. One hemisphere of the brain was fixed in 10% neutral-buffered formalin, and the other was coronally sectioned and frozen at − 80 °C until used.

**Table 2:** Species, age, sex and immunohistochemical results for Aβ and hp-tau in pinnipeds

No	Families	Species	Common name	Age (years)	Sex	Aβ						Hp-tau				
						Plaque					CAA	FC	PC	TC	OC	HP
						FC	PC	TC	OC	HP						
1	Phocidae	*Phoca largha*	Harbor seal	0	M	−	−	−	−	−	−	−	−	−	−	−
2		*Phoca largha*	Harbor seal	3	M	−	−	−	−	−	−	−	−	−	−	−
3		*Phoca largha*	Harbor seal	30	M	−	+	−	−	−	−	+	−	−	−	−
4		*Phoca largha-Phoca vitulina*	Harbor seal	34	F	−	−	−	−	−	−	+	−	−	−	−
5	Otariidae	*Eumetopias jubatus*	Northern sea lion	27	F	+ +	+ +	+	+	−	+	+	−	−	−	−
6		*Zalophus californianus*	California sea lion	27	F	−	−	−	−	−	+ +	+	+	−	−	−
7		*Zalophus californianus*	California sea lion	29	F	+	+	+	+	−	+ +	−	−	−	−	−
8		*Zalophus californianus*	California sea lion	*32*	F	+	+	+	+	+	+ +	+ +	+ +	+	+	−
9		*Neophoca cinerea*	Australian sea lion	32	F	+ +	+ +	+	+	−	+ +	+ +	+ +	+ +	+ +	+ +
10	Odobenidae	*Odobenus rosmarus*	Walrus	35	M	+	+	−	+	−	+	+	−	−	−	−

*M* male, *F* female. Aβ, Hp-tau: −, negative; + , Aβ42-positive plaques, Aβ42 deposition in vascular walls (CAA) and AT8-positive cells in each areas, respectively; +  + , the above lesions were observed severely (Additional file 1: Fig. S1). *FC* frontal cortex, *PC* parietal cortex, *TC* temporal cortex especially in entorhinal cortex, *OC* occipital cortex, *HP* hippocampus

### Histopathology

Formalin-fixed tissues were embedded in paraffin wax and cut into 4- or 8-μm-thick serial sections. Deparaffinized sections were then stained using hematoxylin and eosin (HE), periodic acid-methenamine silver (PAM), Congo red, Thioflavin-S, and the Gallyas-Braak methods.

### Immunohistochemistry

Consecutive sections were stained using an immunoenzyme technique. After deparaffinization and rehydration, antigen retrieval was performed via heating or with formic acid (for Aβ). To deactivate endogenous peroxidase, sections were immersed in 1% hydrogen peroxide in methanol for 5 min. To avoid non-specific binding of the antibody, sections were immersed in 8% skim milk in Tris-buffered saline (TBS). The following primary antibodies were used: mouse anti-Aβ42 (clone 12F4, 1:1000, Millipore, Temecula, CA), rabbit anti-Aβ40 (clone 11A5-B10, 1:1000, Millipore), rabbit anti-AβN1 (1:100, IBL, Gunma, Japan), rabbit anti-AβpN3 (1:100, IBL), rabbit anti-apolipoprotein (Apo) E (1:100, IBL), mouse anti-3-repeat-tau (clone 8E6/C11, 1:100, Millipore), mouse anti-4-repeat-tau (clone 1E1/A6, 1:300, Millipore), mouse anti-hp-tau (Ser202/Thr205) (clone AT8, 1:500, Thermo Scientific, Rockford, IL), mouse anti-hp-tau (Ser212/Thr214) (clone AT100, 1:500, Thermo Scientific), mouse anti-phosphorylated-α-synuclein (Ser129) (clone 81A, 1:150, Abcam, Cambridge, UK), rabbit anti-TDP-43 (1:300, ProteinTech, Rosemont, IL), rabbit anti-GSK-3β (clone 27C10, 1:100, Cell Signaling Technology, Beverly, MA) and rabbit anti-phosphorylated-GSK-3β (Tyr216) (p-GSK-3β (Tyr216)) (1:100, Novus Biologicals, Centennial, CO). After an incubation with each primary antibody at 4℃ overnight, immunolabeled antigens were visualized using the Dako EnVision + System (Dako, Glostrup, Denmark) with 0.02% 3′3-diaminobenzidine plus 0.01% hydrogen peroxide as a chromogen.

### Double-labeling immunofluorescence

To detect the spatial and temporal distribution of Aβ and hp-tau, we performed double immunohistochemistry and double-labeling immunofluorescence. In double immunohistochemistry, sections were initially incubated with the mouse anti-Aβ42 (clone 12F4, 1:1000) or rabbit anti-Aβ42 (1:300, DAKO) antibody and then visualized with 0.02% 3′3-diaminobenzidine plus 0.01% hydrogen peroxide as a chromogen. Slides were incubated with the rabbit anti-GFAP (1:800, DAKO), rabbit anti-Iba-1 (1:200, WAKO, Osaka, Japan), or mouse anti-synaptophysin (clone DAK-SYNAP, 1:500, DAKO) antibody, visualized with new fuchsin (Nichirei Corporation, Tokyo, Japan) as a chromogen, and then counterstained with hematoxylin. In double-labeling immunofluorescence, after an incubation with each of the primary antibodies at 4 °C overnight, sections were washed with TBS, incubated with the corresponding secondary antibodies at room temperature for 1 h, and then mounted with Vectashield (H-1500, Vector Laboratories, Burlingame, CA). The following primary antibodies were used: mouse anti-hp-tau (Ser202/Thr205) (clone AT8, 1:100), rabbit anti-p-GSK-3β (Tyr216) (1:100), rabbit anti-MAP2 (1:100, Millipore), rabbit anti-GFAP (1:400), rabbit anti-Olig2 (1:100, Millipore), and rabbit anti-Iba-1 (1:150). The following secondary antibodies were used: Alexa Fluor 594-conjugated goat anti-mouse IgG (1:100, Invitrogen, Eugene, OR), Alexa Fluor 488-conjugated goat anti-rabbit IgG (1:100, Life Technologies, Eugene, OR), Alexa Fluor 594-conjugated goat anti-rabbit IgG (1:100, Life Technologies), and Alexa Fluor 488-conjugated goat anti-mouse IgG (1:100, Invitrogen). Fluorescent reaction products were optimally visualized using an argon ion laser under a Carl Zeiss LSM700 Confocal Laser Scanning Microscope (Carl Zeiss, Tokyo, Japan).

### Protein extraction

Tissue samples from the forebrain were homogenized in 7.5 volumes of TBS buffer containing 50 mM Tris (pH 7.4), 150 mM NaCl, 1 mM EGTA, 1 mM EDTA, 10.5 μm leupeptin, 307 nM aprotinin, 7.3 μm pepstatin, 1 μM okadic acid, 1 mM glycerophosphate, 1 mM Na_3_VO_4_, and 1 mM NaF. After centrifugation at 23,000 rpm at 4 °C for 15 min, supernatants were collected as TBS-soluble fractions. Pellets were rehomogenized in 7.5 volumes of sucrose buffer containing 0.32 M sucrose, 10 mM Tris/HCl (pH 7.4), 1 mM EGTA, 0.8 M NaCl, 10.5 μm leupeptin, 307 nM aprotinin, 7.3 μm pepstatin, 1 μM okadic acid, 1 mM glycerophosphate, 1 mM Na_3_VO_4_, and 1 mM NaF and then centrifuged as described above. Supernatants were collected and incubated with sarkosyl (Wako, 1% final concentration) at 37 °C for 1 h, followed by centrifugation at 60,000 rpm at 4 °C for 60 min, and then collected as sarkosyl-soluble fractions. Pellets were resuspended in TBS buffer to a volume equivalent to the wet weight of the original tissue (sarkosyl-insoluble fractions). In the dephosphorylation treatment, TBS-soluble fractions were incubated in alkaline phosphatase buffer containing 50 mM Tris–HCl pH 8.0, 100 mM KCl, 1 mM MgSO_4_, 50% glycerol, and 1–3 U bacterial alkaline phosphatase (TaKaRa, Shiga, Japan) at 37 °C for 3 h.

### Western blotting

Protein samples were dissolved in Laemmli Sample Buffer (SB), which included 2-mercaptoethanol, and were then boiled for 10 min. Proteins dissolved in Laemmli SB were separated using a 10% polyacrylamide gel (ATTO, Tokyo, Japan) and then transferred to a nitrocellulose membrane with a pore size of 0.20 μm (GE Healthcare Bio-Sciences AB, Uppsala, Sweden). Non-specific binding was blocked by a treatment with 1% skim milk for 60 min (Nacalai Tesque, Kyoto, Japan). Membranes were probed with the following antibodies at 4 °C overnight: rabbit anti-tau (1:1000, produced by A. Takashima’s laboratory), mouse anti-3-repeat-tau (clone 8E6/C11, 1:500), mouse anti-4-repeat-tau (clone 1E1/A6, 1:500), mouse anti-hp-tau (Ser202/Thr205) (clone AT8, 1:500), mouse anti-hp-tau (Ser212/Thr214) (clone AT100, 1:500), rabbit anti-GSK-3β (clone 27C10, 1:100), rabbit anti-p-GSK-3β (Tyr216) (1:500), and horseradish peroxidase (HRP)-conjugated mouse anti-β-actin (clone 8H10D10, 1:5000, Cell Signaling Technology). After washing the membranes with TBS containing Tween 20, a HRP-conjugated sheep anti-rabbit IgG antibody (1:5000, GE Healthcare UK, Little Chalfont, UK) or HRP-conjugated sheep anti-mouse IgG antibody (1:5000 GE Healthcare UK) was applied. Blots were developed using the ECL Select Western Blotting Detection Reagent (GE Healthcare Bio-Sciences AB). Immunoreactive bands were detected using the ChemiDoc XRS + System (Bio-Rad Laboratories, Hercules, CA).

### Electron microscopy

Formalin-fixed brain samples were washed with phosphate-buffered saline (pH 7.4) and then post-fixed in 1% osmium tetroxide. Samples were dehydrated and embedded in resin Luveak-812 (Nacalai Tesque). Uranyl acetate and lead nitrate-stained ultrathin sections were then examined with a transmission electron microscope (JEM-1400Plus, JEOL, Tokyo, Japan). For negative-stain electron microscopy, samples of sarkosyl insoluble-fractions were stained with uranyl acetate, and examined with the transmission electron microscope.

### RT-PCR analysis

In the molecular analysis of the *APP* and *MAPT* genes, total RNA was extracted from frozen brain tissue using the RNeasy Mini Kit (Qiagen, Valencia, CA). Extracted total RNA was used to synthesize cDNA (Prime Script RT-PCR kit, TaKaRa). Purified cDNA was used for subsequent PCR. Gene-specific primers, listed in Additional file 1: Table S1, were used. PCR was performed as follows: 35 cycles at 98 °C for 10 s and 60 °C for 30 s. PCR products were electrophoresed on an agarose gel and analyzed using a ChemiDocTM imaging system (Bio-Rad Laboratories). Target amplification products were extracted from gel bands using a SV Gel and PCR Clean-Up System (Promega, Madison, WI) and subjected to a sequence analysis (FASMAC, Toyama, Japan). Multiple sequence alignment was performed using CLUSTALW (Kyoto University, Kyoto, Japan).

## Results

### Amyloid β sequence analysis

**Fig. 1:**
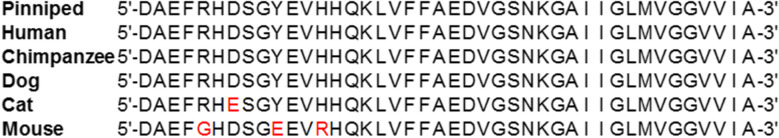
Amino acid sequence analysis of the *APP* gene containing the Aβ region. Multiple Aβ protein alignments among different mammalian orthologs

The sequence analysis of the *APP* transcripts obtained from five pinniped species (case No. 4, 5, 8, 9, and 10) revealed that their Aβ domains had similar nucleic acid sequences. In comparisons with the Phocidae *APP* sequence (case No. 4), the same nucleic acid substitutions in the Aβ domain were found in Otariidae (case No. 5, 8, and 9) and Odobenidae (case No. 10) species (Additional file 1: Fig. S2a). In spite of this nucleic acid substitution in the Aβ domains, the Aβ amino acid sequence was conserved among pinniped species (Additional file 1: Fig. S2b). The multiple sequence alignment of pinniped Aβ with human, chimpanzee, dog, cat, and murine Aβ showed that the amino acid sequence of pinniped Aβ was homologous to that of humans, chimpanzees, and dogs, which formed age-associated amyloid plaques in their brains [1, 10, 17, 18], and different from that of cats and rodents, which did not spontaneously form amyloid plaques [27, 28] (Fig. 1).

### Amyloid plaque formation

**Fig. 2:**
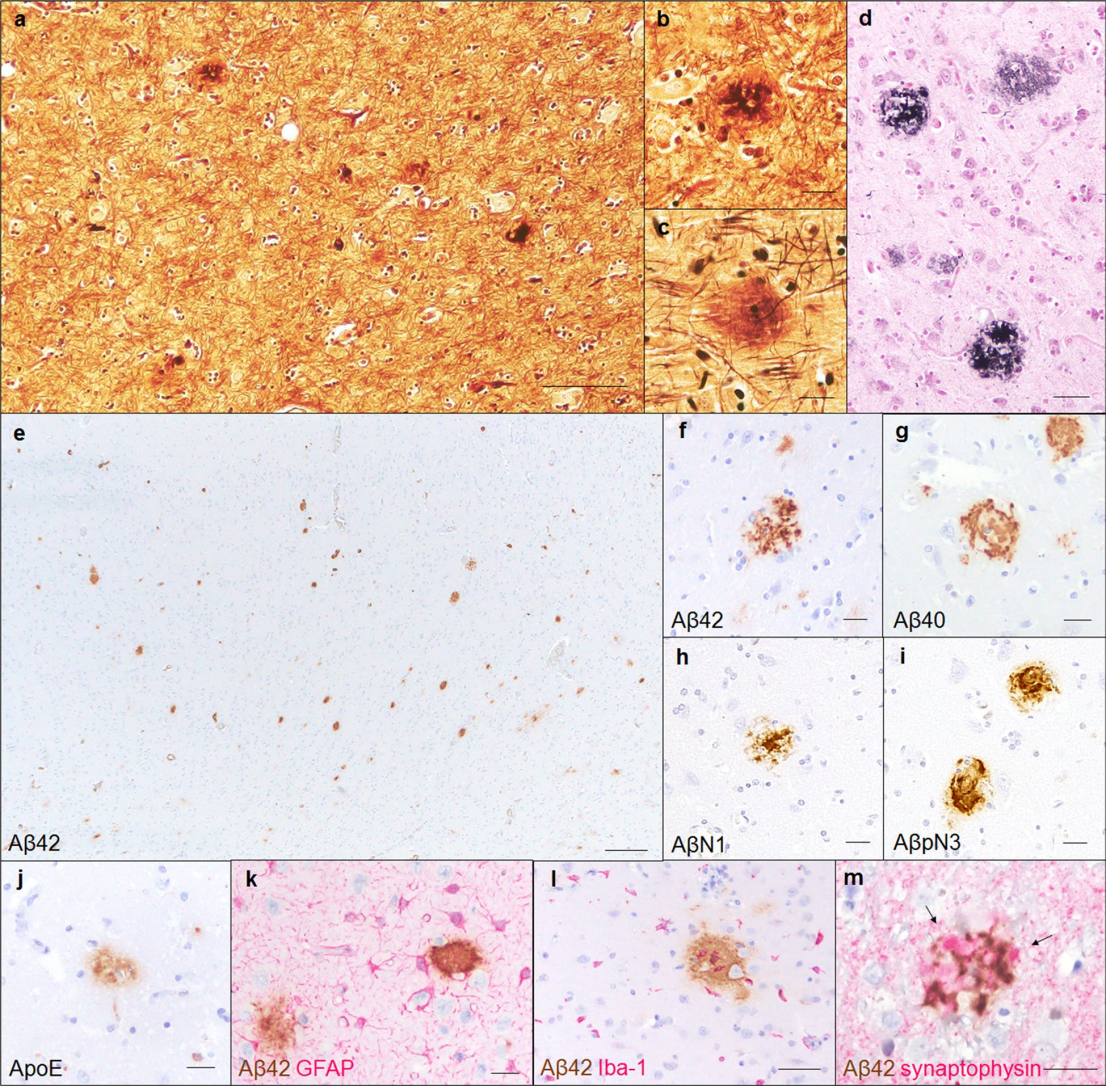
Amyloid plaques in the cerebral cortex of aged pinnipeds (case No. 9). PAM-positive amyloid plaques were observed in the parenchyma of the cerebral cortex (**a**). Amyloid plaques were morphologically classified into two types, small round plaques (**b**) and large plaques (**c**), and were positive for Gallyas-Braak silver staining (**d**). Immunohistochemically, amyloid plaques were positive for Aβ42 (**e**, **f**), Aβ40 (**g**), AβN1 (**h**), AβpN3 (**i**), and ApoE (**j**). The moderate infiltration of GFAP-positive astrocytes (**k**) and Iba-1-positive microglia (**l**) was observed around and on Aβ42-positive amyloid plaques. Swollen dystrophic neurites (arrows) were observed and accompanied by Aβ42-positive amyloid plaques (**m**). Reactions to Aβ42 (**k**, **l**, **m**) are shown in brown, and those to GFAP (**k**), Iba-1 (**l**), and synaptophysin (**m**) in red. Scale bars: 100 μm (**a**); 20 μm (**b**–**d**, **f**–**k**, **m**); 50 μm (**l**); 200 μm (**e**)

Histopathological examinations revealed two types of amyloid plaques in the parenchyma of the cerebral cortex of aged pinniped brains (Fig. 2a). The first type was small round plaques with a PAM-positive central core (Fig. 2b), while the second type was observed as large diffuse plaques without a distinct core (Fig. 2c). These plaques were also visualized using Gallyas-Braak silver staining (Fig. 2d). In an immunohistochemical analysis with anti-Aβ antibodies, numerous Aβ42-positive (Fig. 2e, f) and a few Aβ40-positive deposits (Fig. 2g) were detected in the cerebral cortex of aged pinniped brains. A small proportion of these deposits were positive for AβN1 (Fig. 2h), which recognizes the N terminus of Aβ, and AβpN3 (Fig. 2i), which recognizes N-terminally truncated Aβ. In the cerebral cortex, AβpN3-positive deposits were more common than AβN1-positive deposits. Furthermore, the ApoE protein colocalized with Aβ deposits (Fig. 2j). To elucidate the relationship between aging and amyloid plaque formation, we examined the distribution of Aβ deposits at various ages. In an immunohistochemical analysis with the anti-Aβ42 antibody, although we did not find any Aβ42-positive deposits in infantile (case No. 1, 0 years old) or juvenile brains (case No. 2, 3 years old), Aβ42-positive deposits were detected in the brains of aged pinnipeds (case No. 3–10, 27 to 35 years old) (6/8 cases, Table 2). Aβ42-positive deposits were found in the cerebral cortex of the frontal lobe (5/8 cases), parietal lobe (6/8 cases), temporal lobe (4/8 cases), and occipital lobe (5/8 cases) and also in the hippocampus (only in case No. 8), but not in the cerebral white matter, cerebellum, or brain stem (Table 2). To evaluate peri-plaque neuroinflammation and synaptic changes in aged pinniped brains, we performed double immunohistochemistry. GFAP and Iba-1 staining showed an increased number and size of astrocytes and microglia around Aβ42-positive deposits (Fig. 2k, l). Synaptophysin staining revealed enlarged presynapses surrounding Aβ42-positive deposits, which are considered as small round plaques, showing peri-plaque dystrophies associated with amyloid plaque formation (Fig. 2m).

### Vascular Aβ deposits

**Fig. 3:**
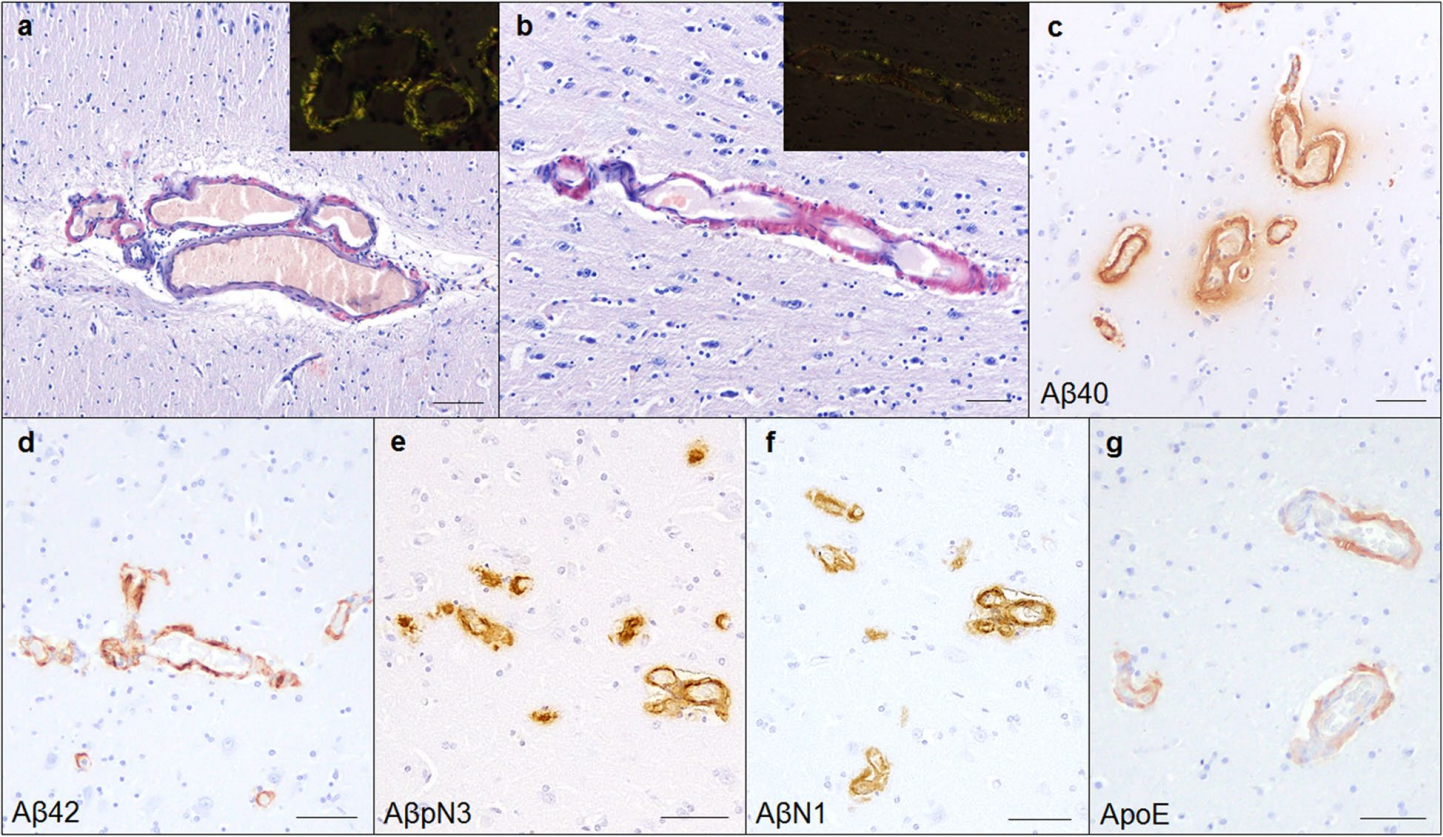
CAA in aged pinniped brains (case No. 9). Congo red-positive amyloid deposition was observed on the blood vessels of the meninges (**a**) and cerebral cortex (**b**). These deposits showed typical apple-green birefringence under polarized light (inset). Immunohistochemically, amyloid deposits of the vessel wall were positive for Aβ40 (**c**), Aβ42 (**d**), AβpN3 (**e**), AβN1 (**f**), and ApoE (**g**). Scale bars: 100 μm (**a**); 50 μm (**b**–**g**)

Histopathologically, eosinophilic deposits were found within the arterial walls of the meninges and the small arterial and capillary walls of the cerebral cortex of aged pinniped brains, which were positive for Congo red staining with apple-green birefringence under polarized light (Fig. 3a, b). In the immunohistochemical analysis with anti-Aβ antibodies, these deposits were positive for Aβ40 (Fig. 3c), Aβ42 (Fig. 3d), AβpN3 (Fig. 3e), and AβN1 (Fig. 3f). The vascular deposition of Aβ42 and Aβ40 was similar, with Aβ42 deposits being located in the vessel wall and Aβ40 deposits surrounding the blood vessels and spreading into the neuropil (Fig. 3c, d). AβN1-positive vessels were more commonly observed than AβpN3-positive vessels. Furthermore, the ApoE protein colocalized with Aβ deposits in the blood vessels (Fig. 3g). In an immunohistochemical analysis with the anti-Aβ42 antibody, vascular Aβ deposits were found in the cerebral cortex and meninges of most aged pinnipeds (6/8 cases), and four aged pinnipeds (case No. 6–9) showed severe Aβ deposition in the blood vessel walls. Aβ42-positive vascular deposits were found in the cerebral cortex of the frontal lobe (6/6 cases), parietal lobe (6/6 cases), temporal lobe (4/6 cases), and occipital lobe (6/6 cases) and also in the hippocampus (5/6 cases), but not in the cerebellum or brain stem (Table 2).

### Expression pattern of tau isoforms

**Fig. 4:**
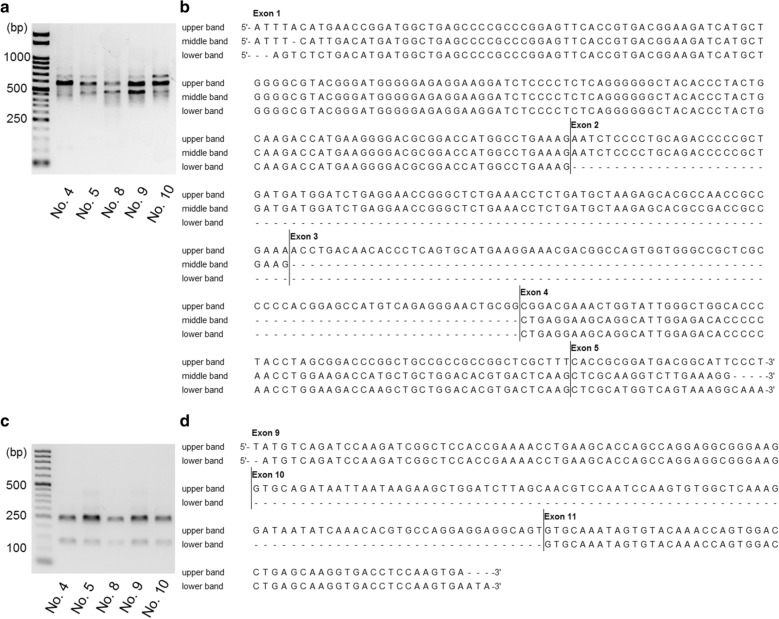
RT-PCR analysis of tau isoforms expressed in pinniped brains. RT-PCR with primer pair 2, which covers exons 0–5, confirmed three types of pinniped *MAPT* mRNA isoforms (**a**) composed of exons 1–5 (upper band), exons 1, 2, 4, and 5 (middle band), and exons 1, 4, and 5 (lower band) (**b**). RT-PCR with primer pair 5, which covers exons 9–11, confirmed two types of pinniped *MAPT* mRNA isoforms (**c**) composed of exons 9–11 (upper band) and exons 9 and 11 (lower band) (**d**)

**Fig. 5:**
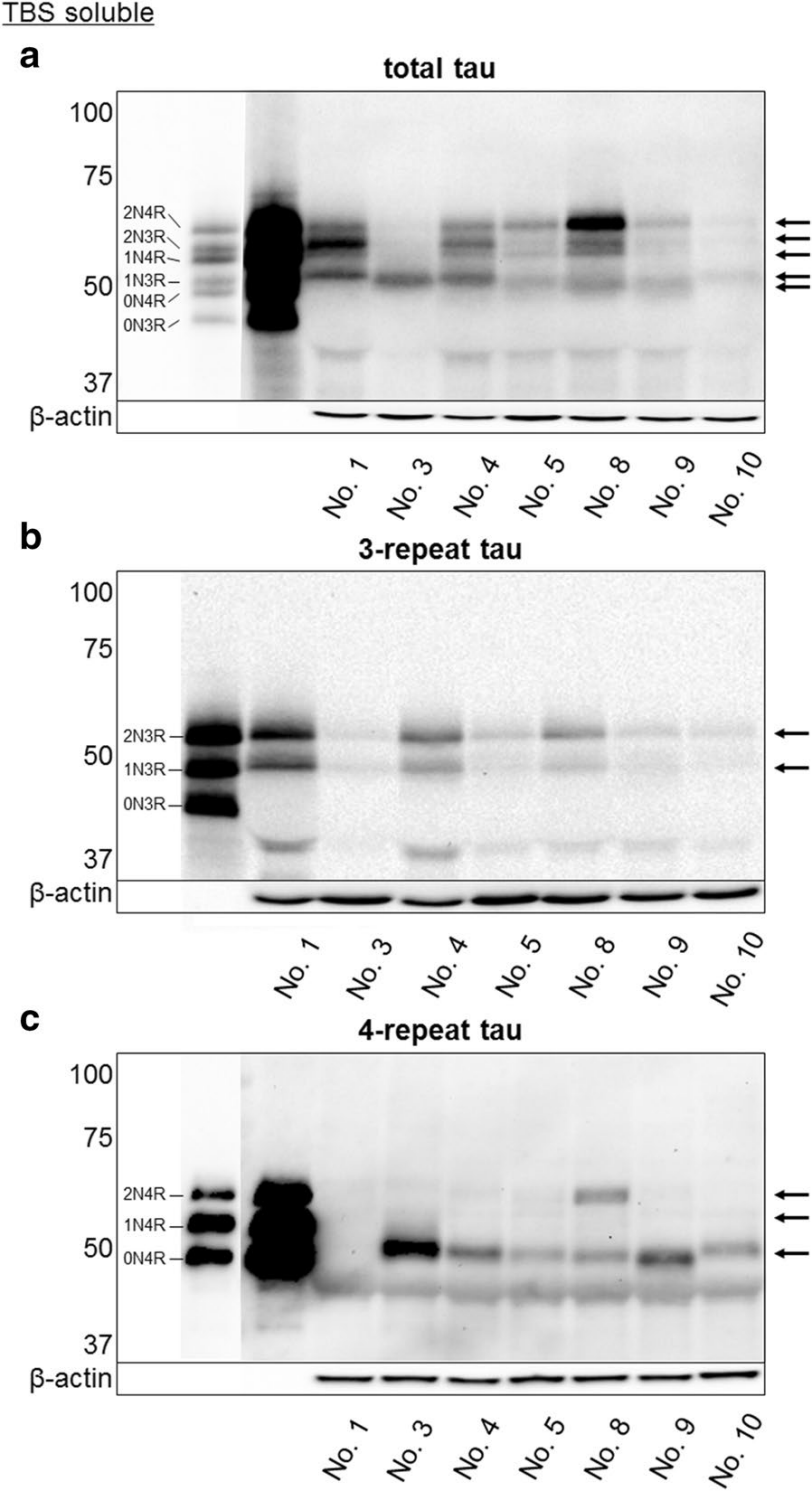
Western blotting analysis of tau isoforms expressed in pinniped brains. Western blotting of TBS-soluble fractions obtained from the forebrains at various ages and treated with alkaline phosphatase. The left lane shows the six isoforms of human tau (recombinantly produced): three 3-repeat tau isoforms (0N3R, 1N3R, and 2N3R) and three 4-repeat tau isoforms (0N4R, 1N4R, and 2N4R). In pinniped brains, five isoforms of tau (1N3R, 2N3R, 0N4R, 1N4R, and 2N4R) were detected using anti-total tau (arrows, **a**), 3-repeat tau (arrows, **b**), and 4-repeat tau antibodies (arrows, **c**)

To confirm the splicing patterns of the pinniped tau protein, *MAPT* transcripts obtained from five pinniped brains (case No. 4, 5, 8, 9, and 10) were examined. RT-PCR with primer pair 2 detected three distinct bands of approximately 450, 550, and 650 bp, and a sequence analysis revealed that all PCR products contained exons 1, 4, and 5 of the *MAPT* gene, and exon 2 was also contained in the 550- and 650-bp bands and exon 3 in the 650-bp band (Fig. 4a, b). RT-PCR with primer pair 3 detected two distinct bands of 350 and 450 bp, and a sequence analysis revealed that all PCR products contained exons 2, 4, 5, and 7 of the *MAPT* gene, and exon 3 was also contained in the 450-bp band (Additional file 1: Fig. S3a, b). RT-PCR with primer pair 4 detected a distinct band at 500 bp, and a sequence analysis revealed that the PCR product contained exons 5, 7, and 9 of the *MAPT* gene (Additional file 1: Fig. S3c, d). RT-PCR with primer pair 5 detected two distinct bands of 150 and 250 bp, and a sequence analysis revealed that all PCR products contained exons 9 and 11 of the *MAPT* gene, and exon 10 was also contained in the 250-bp band (Fig. 4c, d). RT-PCR with primer pair 6 detected a distinct band of 450 bp, and a sequence analysis revealed that all PCR products contained exons 11, 12, and 13 of the *MAPT* gene (Additional file 1: Fig. S3e, f). Exons 1, 4, 5, 7, 9, 11, 12, and 13 of the *MAPT* gene were constitutive exons, and exons 2, 3, 4a, 6, 8, and 10 were subjected to alternative splicing. Exons 4a, 6, and 8 were not present in pinniped brains. Western blotting of TBS-soluble fractions from infantile and aged pinniped cerebrums revealed the expression of five isoforms of tau (1N3R, 2N3R, 0N4R, 1N4R, and 2N4R) (Fig. 5, Additional file 1: Fig. S4).

### NFT formation

**Fig. 6:**
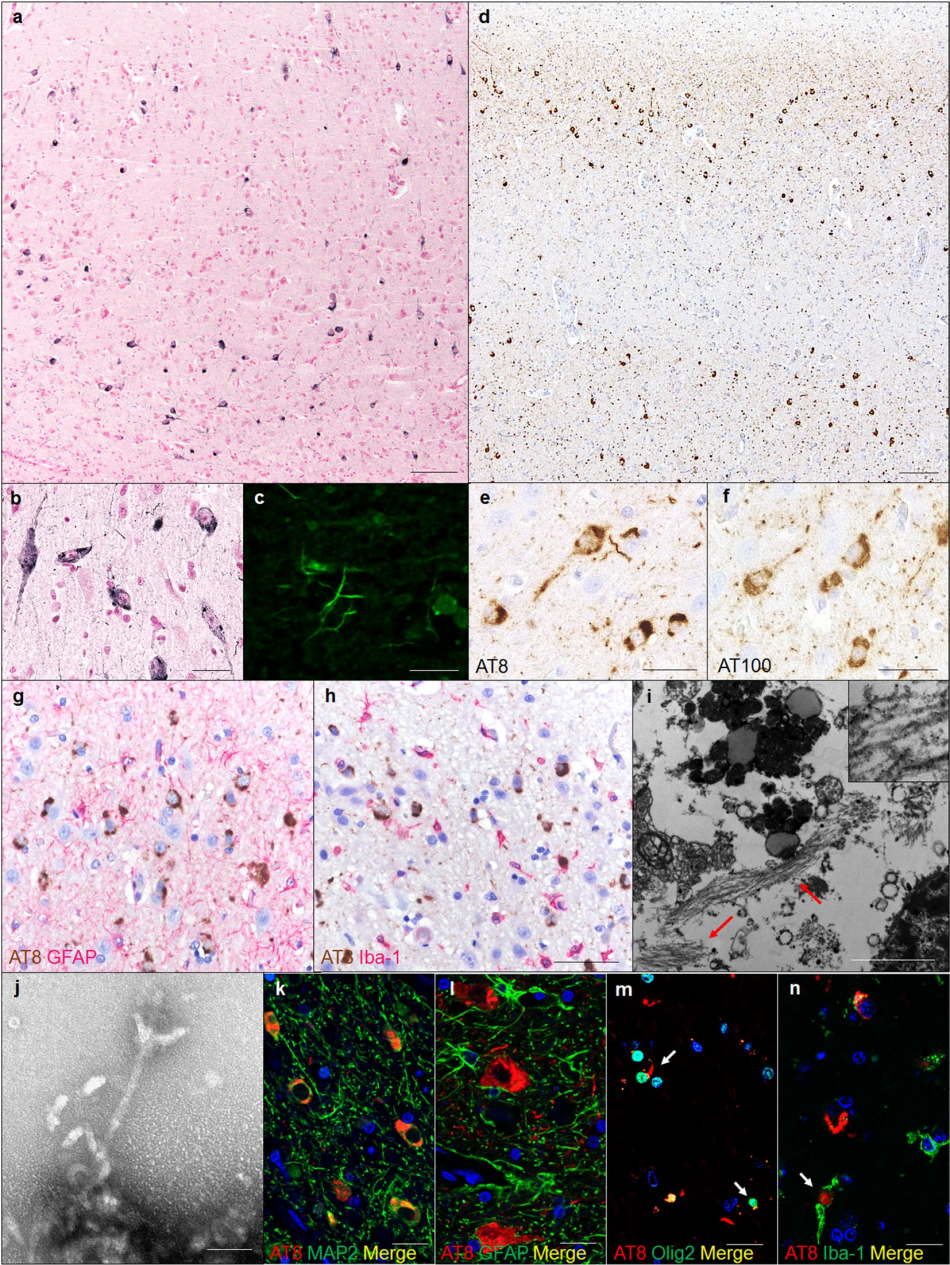
NFTs in the cerebral cortex of aged pinnipeds (case No. 9). Gallyas-Braak staining-positive argyrophilic aggregates were observed in the neuronal somata and neurites in cerebral cortex pyramidal cell layers (**a**, **b**). Thioflavin-S staining-positive neurites were observed in cerebral cortex (**c**). Immunohistochemically, these aggregates were positive for AT8 (**d**, **e**) and AT100 (**f**). GFAP-positive astrocytes (**g**) and Iba-1-positive microglia (**h**) were observed in the cerebral cortex. Reactions to AT8 (**g**, **h**) are shown in brown, and those to GFAP (**g**) and Iba-1 (**h**) in red. Transmission electron microscopy detected bundles of filaments in neuronal soma of aged pinnipeds (arrows, **i**). Sarkosyl-insoluble filaments with a diameter of approximately 10 nm in the cerebral cortex of aged pinnipeds (**j**). AT8-positive hp-tau aggregates were observed in MAP2-positive neurons (**k**), but not in GFAP-positive astrocytes (**l**). Some AT8-positive hp-tau aggregates were observed in Olig2-positive oligodendrocytes (arrows, **m**) and Iba-1-positive microglia (arrow, **n**). Reactions to AT8 (**k-n**) are shown in red, and those to MAP2 (**k**), GFAP (**l**), Olig2 (**m**), and Iba-1 (**n**) in green. Scale bars: 100 μm (**a**, **d**); 50 μm (**b**, **e**–**h**); 2 μm (**i**); 50 nm (**j**); 10 μm (**c**, **k**–**n**)

**Fig. 7:**
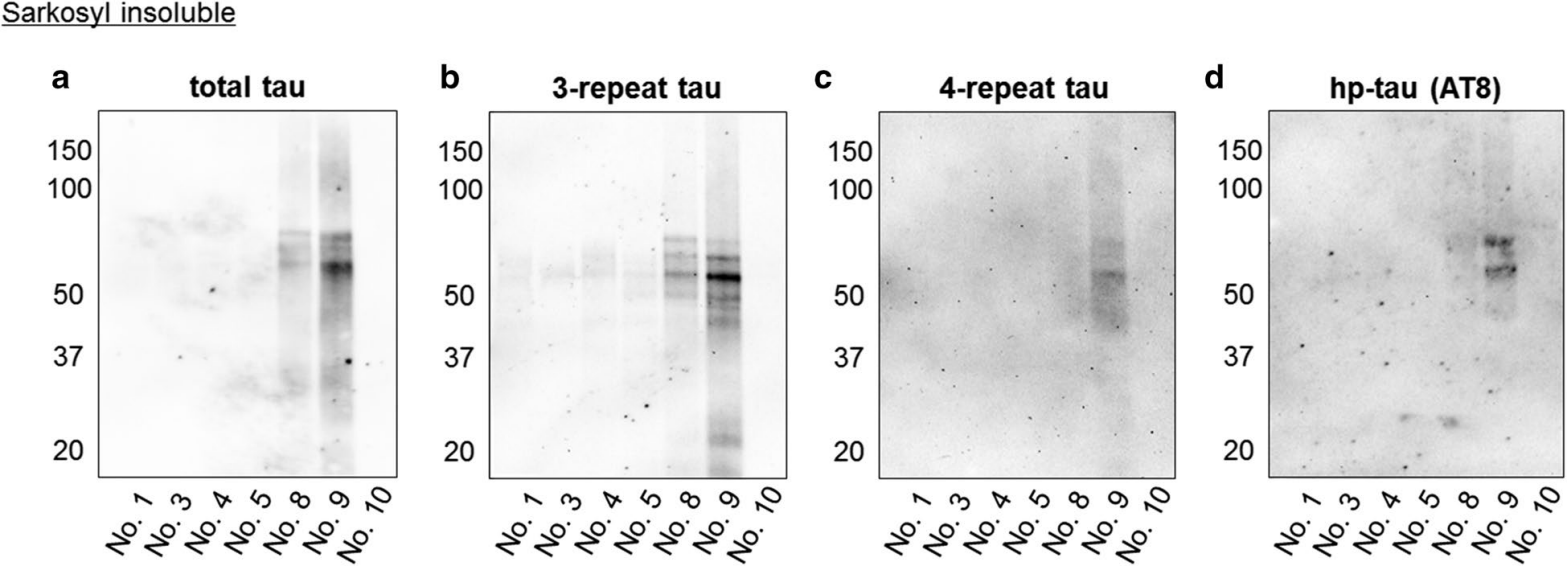
Western blotting analysis of sarkosyl-insoluble fractions obtained from the forebrain of pinnipeds. Sarkosyl-insoluble tau was detected in aged pinniped brains in which abundant hp-tau deposition was confirmed immunohistochemically (case No. 8 and 9) (**a**). Sarkosyl-insoluble tau consisted of 3-repeat (**b**) and 4-repeat tau isoforms (**c**), and was positive for AT8 (**d**)

We examined the tau pathology in aged pinniped brains using Gallyas-Braak silver staining, and confirmed silver-positive fibrillar aggregates in the neuronal somata and neurites of cerebral cortex pyramidal cell layers (layers III and V) (Fig. 6a, b). Thioflavin-S staining detected fibrillar aggregates in the neurites (Fig. 6c). An immunohistochemical analysis with anti-hp-tau antibodies showed that aggregates were positive for AT8 (Fig. 6d, e), which recognized tau phosphorylated at Ser202 and Thr205, and AT100 (Fig. 6f), which recognized tau phosphorylated at Thr212 and Ser214. Immunohistochemistry for GFAP and Iba-1 showed an increased number and size of astrocytes and microglial infiltration in the cerebral cortex of aged pinnipeds (Fig. 6g, h). In electron microscopy, bundles of filaments with a diameter of approximately 10 nm were observed in the neuronal somata and neurites (Fig. 6i). Numerous straight filaments, having a diameter of approximately 10 nm, were detected in sarkosyl-insoluble fractions (Fig. 6j). A double-labeling immunofluorescence analysis revealed that hp-tau aggregates were mostly present in MAP2-positive neuronal cells (Fig. 6k), but not in GFAP-positive astrocytes (Fig. 6l). Additionally, hp-tau aggregates were detected in a few Olig2-positive oligodendrocytes (Fig. 6m) and Iba-1-positive microglia (Fig. 6n). In an immunohistochemical analysis with the anti-AT8 antibody, the majority of pinnipeds older than 27 years old displayed hp-tau aggregates (7/8 cases, Table 2). These aggregates were found in the cerebral cortex, but not in the cerebral white matter, cerebellum, or brain stem. AT8-positive aggregates were observed in the cortex of the frontal lobe (7/8 cases), parietal lobe (3/8 cases), temporal lobe (2/8 cases), and occipital lobe (2/8 cases), and only case No. 9 developed hp-tau aggregates in the hippocampus (Table 2). There was no α-synuclein or TDP-43 pathology in the brains of any pinnipeds (data not shown). The Western blotting analysis using anti-tau antibodies indicated that sarkosyl-insoluble fractions prepared from the frontal lobe cortex tissues of aged pinnipeds contained abundant tau (Fig. 7a). These insoluble tau species, which showed a smear profile in Western blots, consisted of both the 3R and 4R tau isoforms, which were phosphorylated (Fig. 7b-d).

### GSK-3β activation associated with NFT formation

**Fig. 8:**
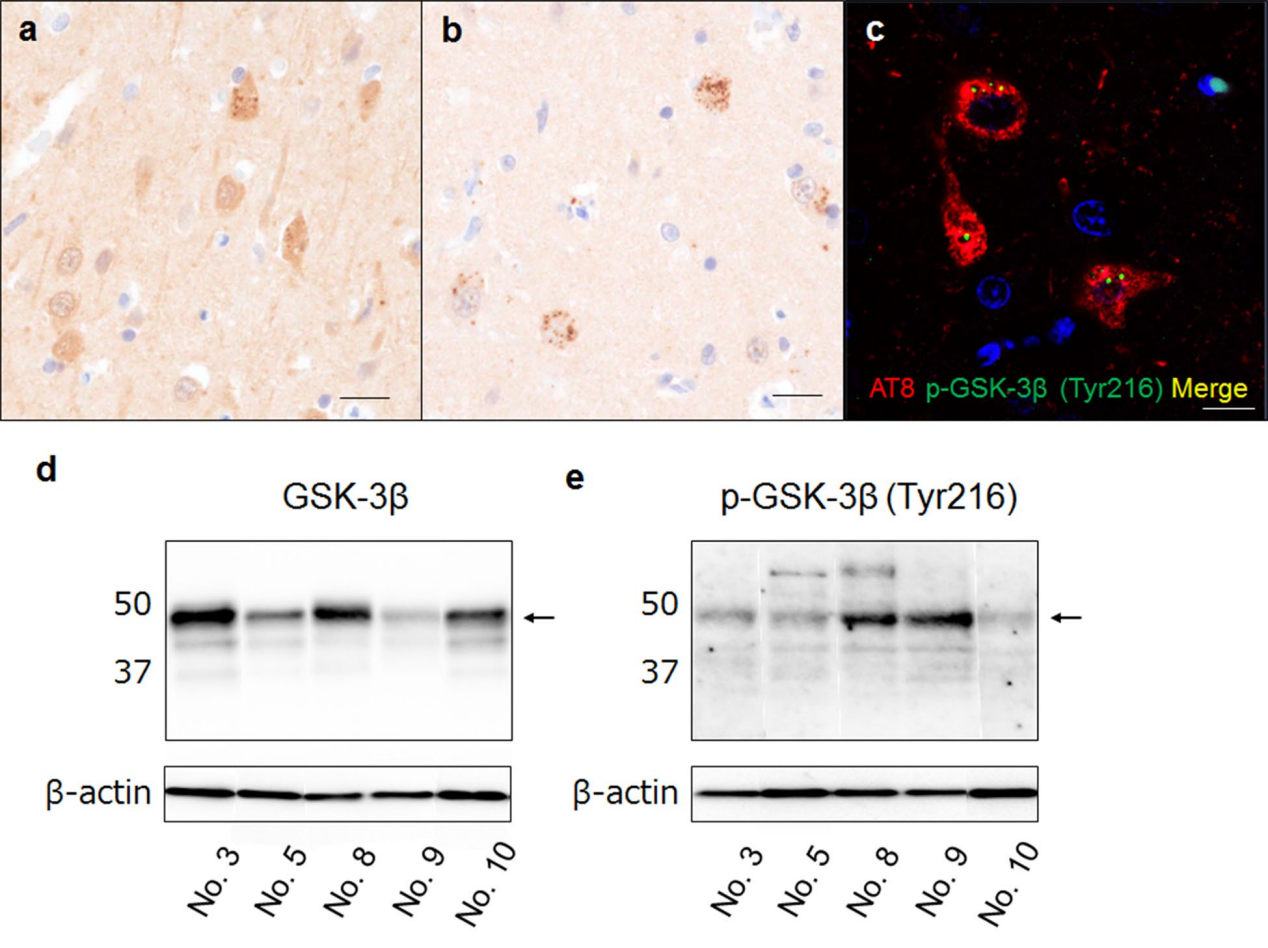
Immunohistochemistry for GSK-3β in pinniped brains (case No. 9). The cytoplasmic uniform distribution of GSK-3β and GSK-3β-positive grains was found in the neuronal somata of aged pinnipeds (**a**). P-GSK-3β (Tyr216)-positive cells were found in aged pinniped brains (**b**). Double-labeling immunofluorescence in the cerebral cortex of aged pinnipeds revealed that p-GSK-3β (Tyr216)-positive grains were localized in AT8-positive cells (**c**) Reactions to AT8 are shown in red, and those to GSK-3β and p-GSK-3β (Tyr216) in green. Scale bars: 20 μm (**a**, **b**); 10 μm (**c**). Western blotting analysis of GSK-3β in TBS-soluble fractions from the forebrain of pinnipeds. A distinct band of GSK-3β with a molecular weight of 46 kDa was detected in pinniped brains (arrow, **d**). A stronger band of p-GSK-3β (Tyr216) was detected in case No. 8 and 9, in which severe hp-tau accumulation was confirmed immunohistochemically, than in other cases (arrow, **e**)

We examined the activation of GSK-3β, a major kinase for tau phosphorylation, in pinniped brains. An immunohistochemical analysis revealed uniform GSK-3β expression in the neuronal cytoplasm of all cases (Fig. 8a). In addition, GSK-3β-positive grains were found in aged cases (Fig. 8a). Furthermore, aged pinnipeds showed p-GSK-3β (Tyr216)-positive grains, which are an active form of GSK-3β, in the neuronal somata of the cerebral cortex (Fig. 8b). A double-labeling immunofluorescence analysis revealed that hp-tau aggregates and p-GSK-3β (Tyr216)-positive grains were detected within the same cells (Fig. 8c). A Western blotting analysis revealed that the expression levels of p-GSK-3β (Tyr216) were higher in case No. 8 and 9, in which severe hp-tau accumulation was confirmed immunohistochemically, than in other cases (Fig. 8d, e).

## Discussion

Animals that spontaneously develop SPs in aged brains, such as monkeys and dogs, share the same Aβ peptide amino acid sequence as humans [29]. In contrast, SP formation has never been demonstrated in non-transgenic wild-type rodents, such as rats and mice, or cats. Rodent Aβ displays three amino acid differences in its N-terminal region from human Aβ [28], and one amino acid located in the N-terminal region of cat Aβ protein differed from that of human Aβ [27]. In the present study, we detected amyloid plaque formation in aged pinniped brains, and their Aβ peptide amino acid sequence was the same as that of human Aβ, providing support for spontaneous amyloid plaque formation specifically developing in animals that express the human type Aβ. In humans, amyloid plaques are generally categorized as SP (neuritic plaque) and diffuse plaques [1]. In the present study, we detected two types of amyloid plaques, small round plaques and large plaques. The small round plaques of pinnipeds were similar to human SPs because of the presence of a PAM-positive central core of Aβ and dystrophic neurites surrounding plaques; however, the large plaques lacking a central core were similar to human diffuse plaques. Previous studies confirmed that human neurotoxic amyloid plaques were predominantly composed of Aβ40 and Aβ42, and Aβ42 was more abundant in plaques than Aβ40 because of its higher rate of fibrillization and insolubility as well as its ability to form insoluble clumps of oligomers [30]. The N-terminal subtype of Aβ peptides also affects their aggregability, and, thus, plaque formation. Aβ truncated at N-terminal position 3 is catalyzed by glutaminyl cyclase, which produces abundant AβpN3 in the AD brain, but not in the normal aging brain [31], and AβpN3 is more depositable than AβN1 [32]. Immunohistochemistry for Aβ revealed that Aβ42 more prominently accumulated than Aβ40 in the parenchyma of pinniped brains, and some amyloid plaques were positive for AβpN3, suggesting the presence of Aβ species with a higher aggregation capability and neurotoxicity in pinniped brains. Though the Aβ42 deposition was severer in the sea lion and walrus brains than seal brains, the distribution pattern was comparable among three pinniped species. Aβ plaques were the most abundant in the frontal, parietal, and temporal neocortices and some were detected in the hippocampus, whereas none were found in the brain stem; this progression aligns with the Thal Aβ phases of deposition in humans [33]. Previous studies revealed that the accumulation of Aβ induced a neuroinflammatory response. The infiltration of microglia and reactive astrocytes is commonly observed in the periphery of amyloid plaques [34], and the barrier formed by astrocytes and microglia around plaques was described as a reactive glial net [35]. In the present study, activated astrocytes and microglia were found in some amyloid plaques (cored plaques), but not in diffuse plaques. In addition to amyloid plaques, we also detected the severe deposition of Aβ in the walls of blood vessels in the cerebral cortex and meninges of aged pinnipeds. Previous human studies reported that CAA was present in more than 80% of patients with AD and up to 40% of cognitively unimpaired elderly individuals [3]. In contrast to amyloid plaques, Aβ40 was more abundant in CAA than Aβ42 [36], and amyloid deposition progressed in a regional pattern, with the greatest accumulation being observed in the occipital cortex followed by the prefrontal cortex and hippocampus. The severity of occipital CAA positively correlated with the severity of AD [37]. In the present study, we detected the vascular deposition of Aβ in 6/8 cases of aged pinnipeds. Its severity was the highest in the frontal, parietal, and occipital regions, followed by the temporal region and hippocampus. These results suggest that pinniped brains frequently develop CAA with aging, with similar progression to that in humans. Although the numbers of Aβ40- and Aβ42-positive blood vessels appeared to be similar, Aβ40-positive dispersed aggregates were also detected at the neuropil around blood vessels in aged pinnipeds, and this distribution pattern has been reported in human patients with dysphoric angiopathy [38]. Based on the perivascular deposition of Aβ40, we speculate that Aβ40 accumulates slightly more than Aβ42 in the vessel walls and is a main component of pinniped CAA.

The activity of the tau protein is influenced by a number of functional tubulin-binding domains and depends on the splicing of exon 10, which directly influences whether the tau protein expressed will contain three (3R) or four (4R) domains, termed incomplete tandem repeats. The existence of two other alternatively spliced exons (exons 2 and 3) increases the maximal number of tau isoforms in the central nervous system to six [39]. The expression patterns of 3R and 4R tau differ among animal species and developmental stages. All six isoforms of tau (0N3R, 1N3R, 2N3R, 0N4R, 1N4R, and 2N4R) are expressed in adult human and cat brains, while 4 isoforms are expressed in adult dog (2N3R, 0N4R, 1N4R, and 2N4R), pig, and bovine brains (1N3R, 2N3R, 1N4R, and 2N4R). Adult rodent brains only express 4R tau isoforms (0N4R, 1N4R, and 2N4R) [40]. Mammalian species that spontaneously develop NFT, such as humans, chimpanzees [14], and cats [17, 22], have been reported to express six tau isoforms in their brains, and the expression of all tau isoforms appears to be a prerequisite for NFT formation. In the present study, we confirmed the alternative splicing of exons 2, 3, and 10 of the *MAPT* gene and the expression of five isoforms of tau in pinniped brains (1N3R, 2N3R, 0N4R, 1N4R, and 2N4R), suggesting that it is sufficient to form NFTs in the absence of 0N3R tau expression in pinniped brains. Tau proteins were hyperphosphorylated and predominantly aggregated in the neurons of the cerebral cortex pyramidal cell layers (layers III and V). These structures were argyrophilic with Gallyas-Braak staining, ultrastructually filamentous, and composed of both 3R and 4R tau isoforms detected in sarkosyl-insoluble fractions. This cytopathology matches NFTs in AD patients [34]; however, the distribution of the tau pathology markedly differed from that in AD patients. Recent studies proposed a seeding hypothesis in which small amounts of misfolded proteins act as seeds that initiate the recruitment of their soluble counterparts into fibrils and the cell-to-cell transmission of protein aggregates, and the spread of NFTs closely correlates with disease progression; therefore, the distribution of NFTs is important for identifying the disease stages of sporadic AD [41]. According to Braak staging, tau inclusions develop in the locus coeruleus as well as in the transentorhinal and entorhinal regions (Stages I and II). This is followed by their presence in the hippocampal formation and some parts of the neocortex (Stages III and IV), followed by large parts of the neocortex (Stages V and VI) [42]. In the present study, though NFTs were more detectable in the sea lion brains than seal and walrus brains, the distribution pattern was comparable among three pinniped species. Pinnipeds did not develop NFTs in the subcortical nuclei, such as the locus coeruleus and magnocellular nuclei. NFTs were predominantly detected within the frontal neocortex in the majority of the aged cases examined (7/8 cases) and in the parietal (3/8 cases) and occipital neocortices (2/8 cases). In severe cases (case No. 8 and 9), the hippocampus and entorhinal cortex were affected as well as entire regions of the cerebral cortex (frontal, parietal, and occipital cortices). These results suggest that tauopathic changes in the aged pinniped brain are characterized by the AD-like formation of NFTs in the neocortex, which originally occurs in the cortex of the frontal lobe and spreads to related cortical regions. Since Aβ plaques were the most abundant in the frontal, parietal, and temporal neocortices, and relatively fewer in the hippocampus, the regional distribution of tau was similar to that of Aβ. Therefore, we speculate that these distribution patterns suggest a relationship between Aβ deposition and hp-tau accumulation in pinniped brains. In the Wnt/GSK-3β hypothesis of AD [43, 44], Aβ has been suggested to inhibit Wnt signaling and induce the activation of GSK-3β, known as Tau kinase I. The activation of GSK-3β induces an increase in the tau pathology associated with decreases and deficits in neurons [45]. We detected the activation of GSK-3β within cells in which hp-tau accumulated in the neocortex of aged pinniped brains, and the amounts of activated GSK-3β were higher in cases with abundant NFTs. Therefore, we speculate that Aβ-induced GSK-3β activation contributes to the formation of NFTs in the neocortex of pinniped and human brains.

Carnivora species have been described with characteristic AD-like pathologies, such as the formation of SPs in aged dogs and bears and NFTs in aged cats. In the present study, we confirmed that aged pinniped species developed SPs and NFTs in the cerebral cortex, and this is the first comprehensive study to report a spontaneous full AD pathology (SP and NFT) in non-primate animals. The simultaneous neocortical distribution of SPs and NFTs and the activation of GSK-3β suggest interactions between Aβ and hp-tau associated with changes in kinase activities in the neocortex of pinniped brains. Although the precise mechanisms underlying pathological differences among animal species currently remain unclear, a basic biochemical difference may be one reason because the Aβ sequence is highly conserved in animals with the spontaneous formation of SPs, such as humans, monkeys, dogs, and pinnipeds, but not mice or cats, while NFTs were detected in animals expressing more than four tau isoforms in their brains, such as humans, monkeys, cats, and pinnipeds, but not mice or dogs (Table 1). Based on these histopathological and biochemical features of pinniped brains, a comparative study among Carnivora species may provide insights into other mechanisms of AD, and may be useful for investigating the pathogenesis of and possible treatments for this disease.

## Supplementary Information

**Additional file 1:** **Table S1.** Primer sequences and expected PCR product sizes (in base pair) used to analyze the expression of APP and tau in pinniped brains. **Fig. S1.** Immunohistochemistry for Aβ42 and AT8 in pinniped brains. **Fig. S2.** Nucleic acid and amino acid sequence analyses of the APP gene containing the Aβ region. **Fig. S3.** RT-PCR analysis of tau isoforms expressed in pinniped brains. **Fig. S4.** A Western blotting analysis of tau isoforms in pinniped brains (longer exposure time of Figure 5C).

## Availability of data and materials

The datasets during and/or analyzed during the current study available from the corresponding author on reasonable request.
