# Supplementary material for: Amyloid β and tau pathology in brains of aged pinniped species (sea lion, seal, and walrus)
Source: Acta Neuropathol Commun. 2021 Jan 7;9:10. doi: 10.1186/s40478-020-01104-3 (PMC7792306; doi:10.1186/s40478-020-01104-3)
Supplement: Supplementary file 1 — Additional file 1: Table S1. Primer sequences and expected PCR product sizes (in base pair) used to analyze the expression of APP and tau in pinniped brains. Fig. S1. Immunohistochemistry for Aβ42 and AT8 in pinniped brains. Fig. S2. Nucleic acid and amino acid sequence analyses of the APP gene containing the Aβ region. Fig. S3. RT-PCR analysis of tau isoforms expressed in pinniped brains. Fig. S4. A Western blotting analysis of tau isoforms in pinniped brains (longer exposure time of Figure 5C). [file 40478_2020_1104_MOESM1_ESM.docx]

**Table S1** Primer sequences and expected PCR product sizes (in base pair) used to analyze the expression of APP and tau in pinniped brains.


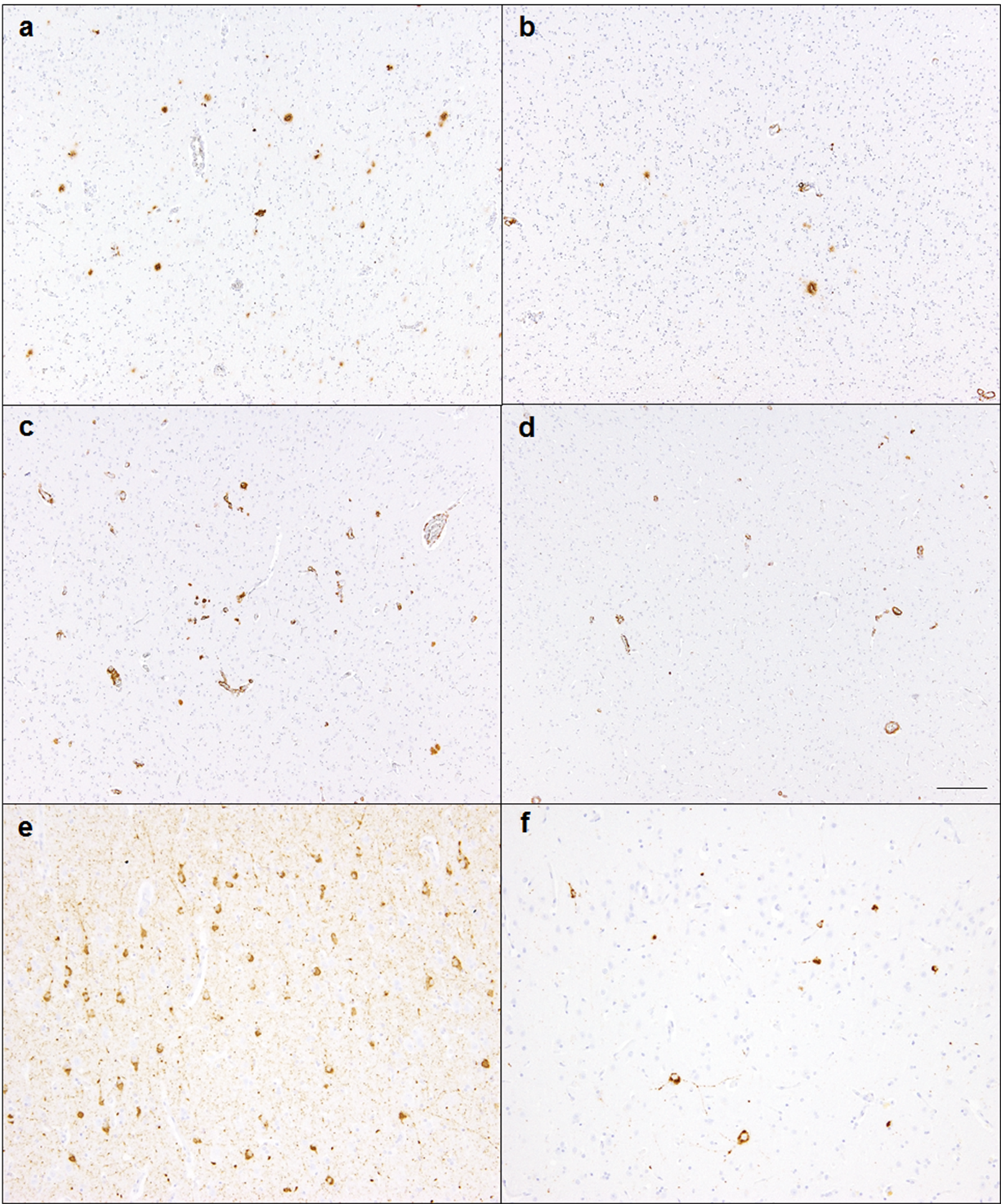


**Fig. S1.** Immunohistochemistry for Aβ42 and AT8 in pinniped brains. In severely affected cases, more than ten Aβ42-positiven plaques (**a**) or twenty Aβ42-positive blood vessels (**c**) were detected in an area of 3.4 mm^2^ or more than thirty AT8-positive cells in an area of 0.55 mm^2^ (**e**). In mildly affected cases, lesions were less apparent (**b**, **d**, **f**). Scale bars: 200 μm (**a**-**d**); 100 μm (**e**, **f**).


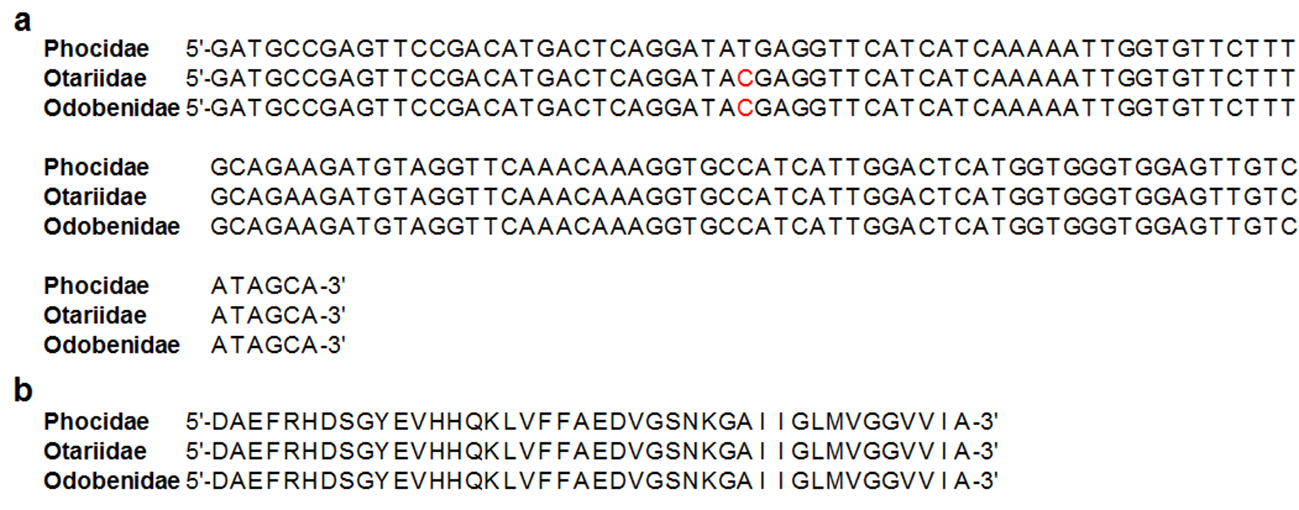


**Fig. S2.** Nucleic acid and amino acid sequence analyses of the *APP* gene containing the Aβ region. Nuclei acid sequences of the pinniped Aβ region (**a**). Amino acid sequences of the pinniped Aβ region (**b**).


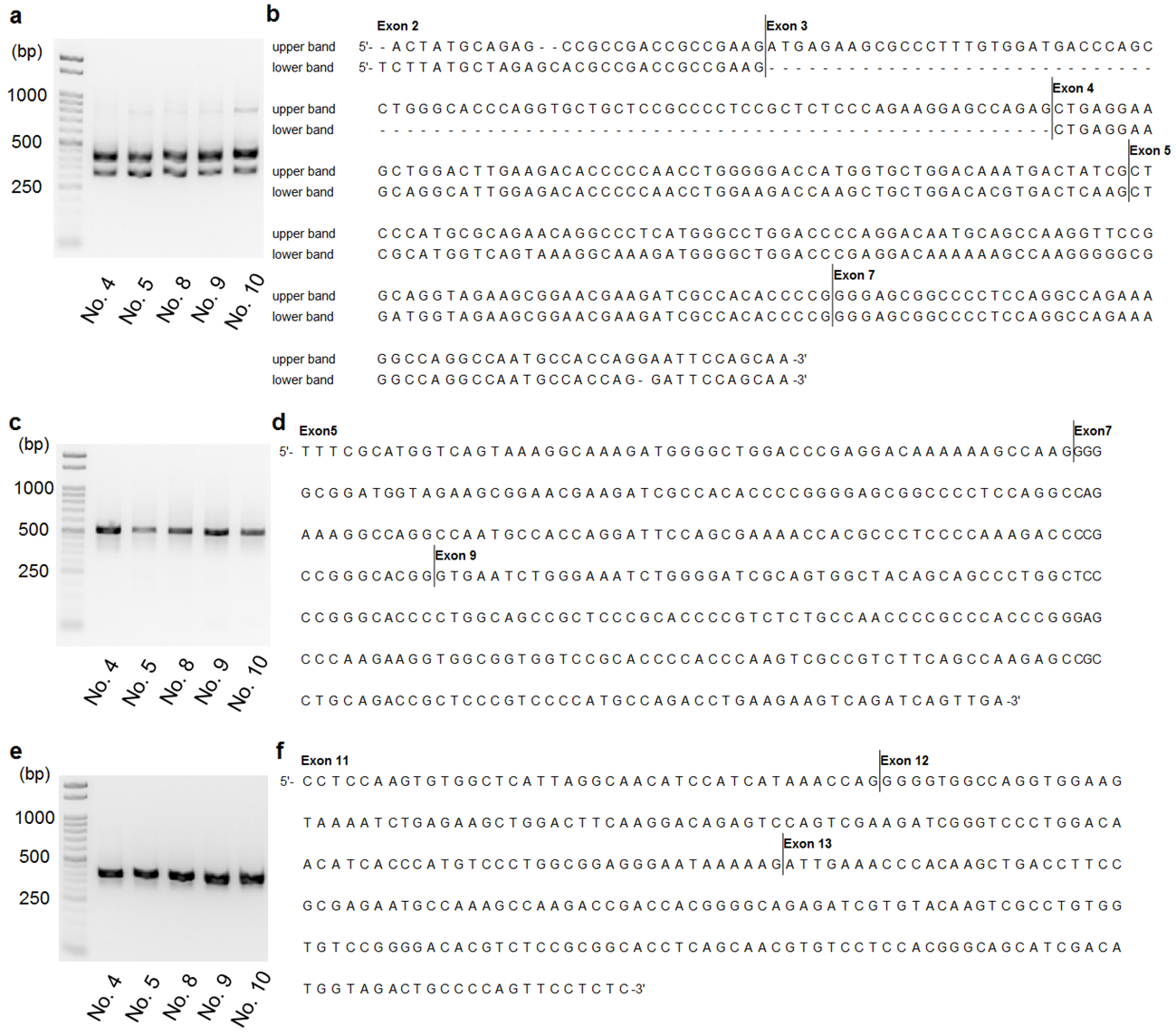


**Fig. S3.** RT-PCR analysis of tau isoforms expressed in pinniped brains. RT-PCR with primer pair 3, which covers exons 2-7, confirmed two types of pinniped *MAPT* mRNA isoforms (**a**) composed of exons 2-5 and 7 (upper band) and exons 2, 4, 5, and 7 (lower band) (**b**). RT-PCR with primer pair 4, which covers exons 5-9, detected a distinct band of pinniped *MAPT* mRNA (**c**) composed of exons 7 and 9 (**d**). RT-PCR with primer pair 6, which covers exons 11-13, detected a distinct band of pinniped *MAPT* mRNA (**e**) composed of exons 11-13 (**f**).


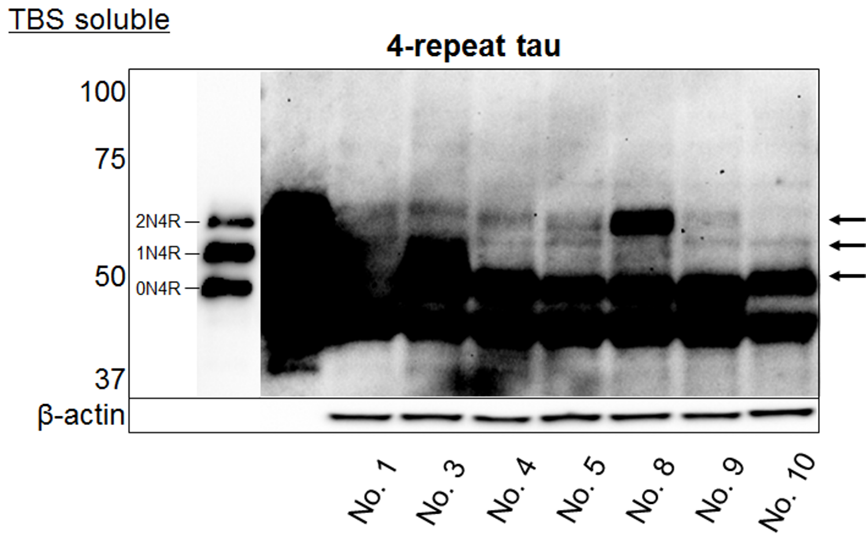


**Fig. S4.** A Western blotting analysis of tau isoforms in pinniped brains (longer exposure time of Figure 5C). Western blotting of TBS-soluble fractions obtained from the forebrain of various ages and treated with alkaline phosphatase. The left lane shows the three isoforms of human tau (recombinantly produced): three 4-repeat tau isoforms (0N4R, 1N4R, 2N4R). In pinniped brains, all three 4-repeat tau isoforms were detected using anti-4-repeat tau antibodies (arrows).
